# Clinical trial effects of acupuncture combined with different general anesthesia on postoperative nausea and vomiting and early recovery after thyroidectomy with intra-operative neuromonitoring: a randomized controlled trial

**DOI:** 10.3389/fmed.2025.1650403

**Published:** 2025-10-16

**Authors:** Weipeng Zhao, Shanshan Jiao, Binglin Peng, Shasha Zhang, Tong Tong, Tao Hu, Na An, Shuyan Wei, Huaqin Liu

**Affiliations:** ^1^Department of Traditional Chinese Medicine, The Fourth Hospital of Hebei Medical University, Shijiazhuang, China; ^2^Department of Anesthesiology, The Fourth Hospital of Hebei Medical University, Shijiazhuang, China; ^3^Department of Anesthesiology, The PLA Bethune International Peace Hospital, Shijiazhuang, China

**Keywords:** acupuncture, propofol anesthesia, thyroidectomy, postoperative nausea and vomiting, recovery quality

## Abstract

**Background:**

Intraoperative neuromonitoring (IONM) is widely used to preserve recurrent laryngeal nerve (RLN) function during thyroid surgery. However, patients undergoing thyroidectomy with IONM may experience a higher incidence of postoperative nausea and vomiting (PONV) and poorer recovery quality. Both acupuncture and propofol anesthesia have been investigated as potential strategies to prevent PONV, but clinical evidence on their combined use in thyroidectomy is limited. This study aimed to evaluate the effects of propofol-based anesthesia combined with postoperative acupuncture on PONV and early recovery within 24 h after thyroidectomy with IONM.

**Methods:**

A total of 135 adult patients were randomly assigned to one of four groups: sevoflurane with sham acupuncture (*n* = 32), sevoflurane with active acupuncture (*n* = 33), propofol with sham acupuncture (*n* = 35), or propofol with active acupuncture (*n* = 35). The primary outcomes were the incidence and severity of PONV and early recovery quality within 24 h. Secondary outcomes included sore throat, headache, and dizziness.

**Results:**

Compared with sevoflurane, propofol anesthesia significantly reduced PONV severity (OR = 0.313; 95% CI, 0.123–0.797; *p* = 0.014) and the frequency of postoperative vomiting (OR = 0.329; 95% CI, 0.115–0.939; *p* = 0.038). Active acupuncture also significantly reduced PONV severity compared with sham acupuncture (*p* = 0.008). However, combining propofol anesthesia with active acupuncture did not provide additional benefits beyond each intervention alone.

**Conclusion:**

Both propofol anesthesia and postoperative acupuncture independently reduced the incidence and severity of PONV after thyroidectomy with IONM. No synergistic effect was observed, possibly due to mechanistic differences or the limited intensity of the acupuncture protocol. Further research is warranted to optimize acupuncture parameters and clarify the clinical utility of combined approaches across broader surgical settings.

**Clinical trial registration:**

The clinical trial was registered in the Chinese Clinical Trial Registry under the identifier ChiCTR2400082127.

## 1 Introduction

PONV is a common and distressing complication after thyroidectomy, with an incidence of 20–60% despite the use of prophylactic anti-emetics (1, 2). Beyond impairing comfort and satisfaction, PONV may precipitate serious consequences, including postoperative hemorrhage, delayed recovery, and increased healthcare costs (3, 4).

IONM is widely adopted during thyroid surgery to reduce the risk of RLN injury (5). However, IONM has been associated with a higher incidence of PONV (5, 6), likely reflecting anesthetic adjustments made to optimize monitoring—such as minimizing neuromuscular blockade, increasing opioid use to prevent movement, and prolonging operative time. Although guidelines for PONV prevention are available, their effectiveness in routine practice remains suboptimal (7). In addition, the limited efficacy and potential adverse effects of antiemetic drugs underscore the need for safer, non-pharmacological strategies.

Propofol-based TIVA, which has intrinsic antiemetic properties, is recommended for patients at high risk of PONV (7). Acupuncture has also attracted interest as a non-pharmacological intervention for postoperative gastrointestinal symptoms (8, 9). Common sensations during acupuncture—such as soreness, heaviness, or numbness at the stimulation site—are regarded by some practitioners as markers of physiological response and have been explored as behavioral indicators in research (8, 9). Stimulation at Neiguan (PC6), Zusanli (ST36), and Zhongwan (RN12) has been reported to alleviate nausea and gastrointestinal discomfort (10). These acupoints were selected according to traditional Chinese medicine theory; while prior trials support their clinical use, putative mechanisms remain largely theoretical and lack validation by modern biomedical research. Notably, clinical evidence on combining acupuncture with propofol-based anesthesia during thyroidectomy is limited.

This study aimed to evaluate whether propofol-based anesthesia combined with postoperative acupuncture reduces the incidence of PONV and improves early postoperative recovery in patients undergoing IONM-assisted thyroidectomy. We also explored whether the combination confers any additional benefit compared with either intervention alone.

## 2 Methods

### 2.1 Study design and ethical approval

This single-center, randomized, controlled trial evaluated the effects of propofol-based anesthesia combined with active acupuncture on nausea, vomiting, and early recovery quality after thyroid surgery. The protocol was approved by the Ethics Review Committee of the Fourth Hospital of Hebei Medical University (approval No. 2024012) and registered in the Chinese Clinical Trial Registry (ChiCTR2400082127). Written informed consent was obtained from all participants before enrollment.

### 2.2 Patient selection

Adults aged 18–80 years with ASA physical status I–III scheduled for elective thyroidectomy were eligible. Exclusion criteria were infection or eczema over acupoints; cervical vertebral disease; motion sickness or vertebrobasilar insufficiency; history of opioid use; severe renal or hepatic dysfunction; coagulopathy or other hematologic disease; pregnancy or breastfeeding; and allergy to any study medication.

### 2.3 Randomization and group allocation

Participants were randomly assigned (1:1:1:1) using a computer-generated sequence to one of four groups: sevoflurane + sham acupuncture (*n* = 32), sevoflurane + active acupuncture (*n* = 33), propofol + sham acupuncture (*n* = 35), or propofol + active acupuncture (*n* = 35). Randomization was stratified by anesthesia type (sevoflurane vs. propofol) and acupuncture (active vs. sham). Allocation was concealed with sealed, opaque envelopes, opened immediately before induction of anesthesia and initiation of acupuncture, respectively.

### 2.4 Anesthesia protocol

Anesthesia was induced with intravenous sufentanil (0.5–0.6 μg·kg^−1^), remimazolam (0.3 mg·kg^−1^), and cisatracurium (0.05 mg·kg^−1^), followed by tracheal intubation 7 min after supraglottic topical application of 4 mL 2% lidocaine. Maintenance was either inhaled sevoflurane (1.5–2.0%) in 50% oxygen/air or intravenous propofol (6–10 mg·kg^−1^·h^−1^), titrated to a bispectral index of 40–55. Remifentanil (0.1–0.2 μg·kg^−1^·min^−1^) was administered for analgesia. Pharmacologic antagonism for neuromuscular recovery was not routinely used. For postoperative analgesia, flurbiprofen axetil (50 mg) was given 30 min before the end of surgery. Tracheal extubation was performed in the operating room or post-anesthesia care unit when spontaneous breathing was adequate and SpO₂ exceeded 90%.

### 2.5 Acupuncture intervention

Participants received active or sham acupuncture 10 min to 2 h after returning to the ward. Treatments were delivered by a single experienced acupuncturist. Acupoints included bilateral Neiguan (PC6), Zusanli (ST36), Fengchi (GB20), Tianrong (SI17), and Zhongwan (RN12) (10–14). We selected a PC6–ST36–GB20 montage to target core mechanisms and common accompaniments of PONV: PC6 is the best-evidenced antiemetic point, acting via vagal–nucleus tractus solitarius pathways and modulation of gastric rhythm and 5-HT3–related signaling (15, 16); ST36 augments vagal tone/gastrointestinal motility and improves gastric slow waves/HRV (16, 17); GB20 addresses vestibular–occipital circuitry relevant to postoperative dizziness/headache (18).

After skin antisepsis, sterile filiform needles (0.25 mm; Hwato, 0.25 × 40 mm or 0.25 × 25 mm) were inserted vertically at PC6 (10–20 mm) and ST36 (20–30 mm), and obliquely antero-medially at GB20 (10–15 mm). Mild manual stimulation (de-qi) for 10–15 s every 10 min was applied; retention 25–30 min covering emergence. Intensity was kept below the movement threshold. Predefined stopping rules included movement, discomfort, bleeding, or monitoring interference. All patients were supine. Sham acupuncture used validated blunt-tipped needles in guide tubes at the same acupoints, mimicking penetration without skin puncture, to reproduce the tactile experience without physiological stimulation.

Reporting standards: The intervention follows STRICTA 2010 (CONSORT extension) and TIDieR recommendations, detailing point selection/rationale, needling (location, depth/angle, response sought), regimen (dose, timing, frequency), practitioner background, and control procedures (19–21). See Supplementary Table S_STRICTA.

### 2.6 Blinding procedures

Participants in each group received active or sham acupuncture individually in a private room and were instructed not to communicate with other participants or disclose allocation to outcome assessors. All acupuncture procedures were performed by a single experienced acupuncturist who was not involved in postoperative assessments. A trained research assistant, blinded to allocation, conducted bedside evaluations 24 h after surgery.

Because full participant blinding in acupuncture studies is inherently challenging, a standardized sham procedure was used to simulate the tactile experience of real needling without physiological stimulation. In the sham group, modified blunt-tipped needles were applied at the same acupoints using validated guide tubes; these devices mimicked needle penetration without skin puncture, minimizing sensory differences between sham and active interventions.

### 2.7 Outcome measures

The primary outcomes were the occurrence and severity of PONV within the first 24 h after surgery, as well as the quality of early recovery at 24 h. The time of return to the ward was defined as time 0. PONV occurrence was defined as any episode of nausea, retching, or vomiting, with vomiting counted irrespective of nausea. PONV severity was assessed using a 0–10 numeric rating scale (NRS; 0 = none, 10 = worst imaginable) and categorized into bands of 1–3, 4–6, and 7–10. Early recovery was evaluated with a 0–70 short-form recovery score, constructed from seven items derived from the QoR-15 (breathing comfort, appetite, energy, sleep quality, self-care/hygiene, communication/voice, and sadness/depression). This instrument is not equivalent to the standard 15-item QoR-15 (0–150). Full item wording, anchors, and scoring rules are provided in Supplementary Table Sx.

Secondary outcomes within the first 24 h included sore throat, headache, dizziness, and sleep quality on the night of surgery, and intra-operative opioid consumption (expressed as intravenous morphine-equivalent dose). Sore throat was rated on a four-point ordinal scale (0 = none; 1 = mild, reported only when questioned; 2 = moderate, reported spontaneously without prompting; 3 = severe, accompanied by hoarseness or voice change). Headache was assessed on a 0–10 NRS and similarly archived in bands (1–3, 4–6, 7–10), then classified as none, mild, moderate, or severe, with severe defined as NRS ≥ 7. Dizziness was recorded as a binary yes/no outcome. Sleep quality was assessed on a four-point scale (0 = better than at home; 1 = similar to at home; 2 = slightly worse; 3 = much worse).

### 2.8 Sample size calculation

**Figure 1:**
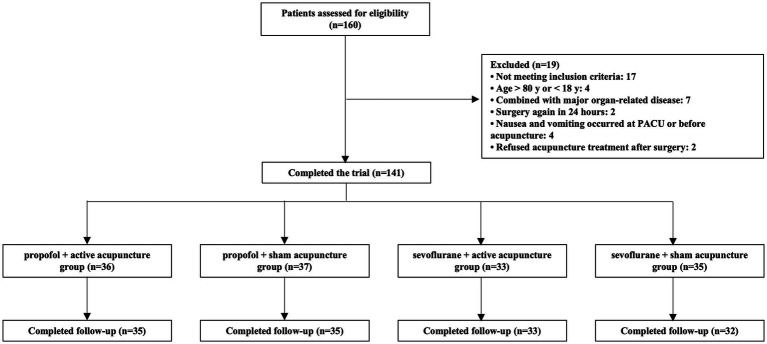
Study flowchart. A total of 160 patients were assessed for eligibility. After exclusions, 141 participants were enrolled and randomized into four groups: propofol + real acupuncture (*n* = 36), propofol + sham acupuncture (*n* = 37), sevoflurane + real acupuncture (*n* = 33), and sevoflurane + sham acupuncture (*n* = 35). Final follow-up was completed for 135 participants.

This was a 2 × 2 factorial trial with equal allocation to four arms (propofol + active acupuncture, propofol + sham acupuncture, sevoflurane + active acupuncture, and sevoflurane + sham acupuncture). Based on routine practice, the sevoflurane-based regimen was associated with an incidence of any PONV of approximately 58% in thyroidectomy with IONM. We hypothesized that the combined intervention of propofol-based anesthesia plus active acupuncture would reduce the incidence of any PONV to 30%, corresponding to an absolute reduction of 28% relative to the most emetogenic arm. Using a two-sided *α* of 0.05 and 80% power, this difference required 29 patients per arm (total 116). Allowing for about 10% attrition, the target sample size was 129 participants (≈32 per arm). Ultimately, 160 patients were screened and 135 participants were included in the final analysis (Figure 1). Under the standard no-interaction assumption for factorial designs, this sample size also provided adequate power to detect main effects of comparable magnitude. Baseline characteristics were summarized as mean (SD) or median [IQR] for continuous variables and n (%) for categorical variables, with between-group balance shown in Table 1.

**Table 1:** Baseline demographic and clinical characteristics of patients data are presented as mean ± SD or number (percentage), as appropriate.

Characteristic	RP (*n* = 35)	SP (*n* = 35)	RS (*n* = 33)	SS (*n* = 32)	All participants (*n* = 135)
Gender, *n*(%)					
Male	10(28.57)	4(11.43)	4(12.12)	10(31.25)	28(20.74)
Female	25(71.43)	31(88.57)	29(87.88)	22(68.75)	107(79.26)
Mean age (SD), *y*	45.57(11.64)	44.06(10.81)	50(11.83)	44.94(11.60)	46.11(11.57)
Mean BMI(SD), *kg/m^2^*	25.43(2.30)	24.54(3.08)	24.88(2.59)	24.69(2.89)	24.89(2.72)
ASA, *n*(%)					
ASA II	34(97.14)	35(100)	32(96.97)	32(100)	133(98.52)
ASA III	1(2.86)	0(0)	1(3.03)	0(0)	2(1.48)
Postoperative pathological types *n*(%)					
Papillary thyroid carcinoma	32(91.43)	29(82.86)	26(78.79)	25(78.13)	112(82.96)
Nodular goiter with adenomatous hyperplasia	1(2.86)	6(17.14)	7(21.21)	5(15.62)	19(14.07)
Hashimoto’s thyroiditis	2(5.71)	0(0)	0(0)	2(6.25)	4(2.97)

BMI = body mass index; ASA = ASA physical status classification system, ASA II: a patient with mild systemic disease; ASA III: a patient with severe systemic disease; Papillary thyroid carcinoma = the most common type of thyroid cancer; Nodular goiter with adenomatous hyperplasia = it is the category of thyroid adenoma, which is a benign tumor; Hashimoto’s thyroiditis = a chronic autoimmune inflammation of the thyroid gland.

### 2.9 Statistical analysis

Prespecified factorial models were used for the primary outcomes, including the main effects of anesthesia (propofol vs. sevoflurane) and acupuncture (active vs. sham), as well as the anesthesia × acupuncture interaction. Main effects were estimated from reduced models without the interaction unless otherwise specified, and the interaction was tested using a Wald term. Any PONV and vomiting ≥1 episode were analyzed with logistic regression; PONV severity was treated as an ordered outcome using proportional-odds models; and the 24-h early recovery score (0–70) was analyzed with linear models. For secondary outcomes, sore throat (0–3) and headache (NRS archived into bands of 1–3, 4–6, and 7–10) were first analyzed as binary outcomes (any vs. none) using logistic models, while dizziness was analyzed as yes/no. As sensitivity analyses, ordered severity distributions (none/mild/moderate/severe) for sore throat and headache were compared using the Mann–Whitney rank-sum test. Intra-operative opioid consumption was compared among groups using ANOVA or Kruskal–Wallis tests as appropriate.

To complement the factorial analysis, omnibus and multiplicity-adjusted pairwise tests were performed across the four arms (propofol + active, propofol + sham, sevoflurane + active, and sevoflurane + sham). ANOVA was used for continuous outcomes (0–70 score), Kruskal–Wallis for ordered outcomes (PONV severity and sore throat), and chi-square or Fisher’s exact test for binary outcomes (any PONV, vomiting ≥1, sore throat, headache, and dizziness). Pairwise contrasts were adjusted using the Holm step-down procedure. Full results, including effect sizes, 95% confidence intervals, and adjusted *p* values, are provided in Supplementary Tables S1–S3; factorial models remain the primary analysis. Effect sizes are presented as adjusted odds ratios (aORs) with 95% confidence intervals and exact *p-*values for binary outcomes, and as mean differences with 95% confidence intervals for continuous outcomes. Two-sided *α* = 0.05 defined statistical significance. Analyses were performed using SPSS version 26.

Equation 1 (binary logistic model; any PONV, vomiting ≥1, sore throat, headache, dizziness):


$$\begin{array}{l} \text{logit}\{P(Y=1)\}=\beta_{0}+\beta_{1}I(\text{propofol})+\beta_{2}I(\text{active acupuncture})\\ +\beta_{3}I(\text{propofol}\times \text{active}) \end{array}$$

The reference levels were sevoflurane and sham acupuncture; odds ratios were calculated as exp.(*β*), with values <1 indicating a protective effect of propofol or active acupuncture when the outcome (Y) denoted an adverse event. Post-hoc power was also computed for each factorial term using the observed coefficient and standard error with a Wald noncentral χ^2^ approximation (α = 0.05), and results are presented in Supplementary Table S4.

## 3 Results

Between April 9 and June 28, 2024, 160 patients were screened; 17 failed eligibility and 2 declined postoperative acupuncture. Six participants were lost to follow-up, leaving 135 participants who completed the trial (propofol + active *n* = 35; propofol + sham *n* = 35; sevoflurane + active *n* = 33; sevoflurane + sham *n* = 32) (Figure 1). Baseline characteristics were well balanced across groups (Table 1).

For the primary outcomes, the four-arm risks of any PONV were 78.1% (25/32) for sevoflurane + sham, 60.6% (20/33) for sevoflurane + active, 45.7% (16/35) for propofol + sham, and 28.6% (10/35) for propofol + active. In factorial models, propofol compared with sevoflurane reduced the risk of any PONV (aOR 0.25, 95% CI 0.12–0.52; *p* = 0.0002) and vomiting ≥1 episode (aOR 0.329, 95% CI 0.115–0.939; *p* = 0.038), and was associated with lower PONV severity (proportional-odds aOR 0.313, 95% CI 0.123–0.797; *p* = 0.015). Active compared with sham acupuncture also reduced any PONV (aOR 0.45, 95% CI 0.22–0.95; *p* = 0.035). No anesthesia × acupuncture interaction was detected for any PONV endpoints (Wald *p* = 0.897), indicating additive rather than synergistic effects (Table 2).

**Table 2:** Primary outcomes at 24 h — prespecified 2 × 2 factorial models.

Outcome	Propofol vs. Sevoflurane (aOR, 95% CI)	*p*	Active vs. Sham (aOR, 95% CI)	*p*	Interaction (Wald *p*)
Any PONV (yes/no)	0.248 (0.119–0.518)	0.0002	0.454 (0.218–0.946)	0.0349	0.897
Vomiting ≥ 1 episode	0.258 (0.120–0.552)	0.0005	1.116 (0.528–2.360)	0.7735	0.811
PONV severity (ordinal 0–3)	0.313 (0.123–0.797)	0.0149	0.216 (0.083–0.567)	0.0018	0.956

Reference levels are sevoflurane and sham acupuncture. For adverse outcomes (any PONV, vomiting ≥1, higher severity), values <1 favor propofol and/or active acupuncture. Interaction p values are from the corresponding full model with the product term. aOR = adjusted odds ratio; CI = confidence interval; PONV = postoperative nausea and vomiting.

Early recovery at 24 h, measured by the 0–70 short-form score (higher = better), did not differ by anesthesia (mean difference [propofol–sevoflurane] + 1.39, 95% CI − 0.75 to +3.54; *p* = 0.204) or by acupuncture (active–sham +1.34, 95% CI − 0.80 to +3.48; *p* = 0.221); the interaction term was non-significant (*p* = 0.553). Medians [IQR] were 64 [60–67] vs. 62 [58–67] for propofol vs. sevoflurane and 62.5 [59–68] vs. 63 [58–66] for active vs. sham (Table 3).

**Table 3:** Early recovery at 24 h — 0–70 short-form recovery score (higher = better).

Arm	*n*	Mean ± SD	Median [IQR]
Propofol + Active	35	63.2 ± 6.4	64 [62–68]
Propofol + Sham	35	62.5 ± 6.0	64 [60–67]
Sevoflurane + Active	33	62.4 ± 5.7	62 [58–68]
Sevoflurane + Sham	32	60.4 ± 7.3	62 [55–66]

Factorial model estimates (linear models)		
Contrast	Mean difference (95% CI)	*p*
Propofol – Sevoflurane	+1.39 (−0.75 to 3.53)	0.204
Active – Sham acupuncture	+1.34 (−0.80 to 3.48)	0.221

Interaction (anesthesia × acupuncture): *p* = 0.553. The 0–70 short form is a non–QoR-15 instrument constructed from seven items; higher scores indicate better recovery. IQR = interquartile range; CI = confidence interval.

Regarding secondary outcomes within 24 h, sore throat (any vs. none) showed no significant differences (propofol vs. sevoflurane aOR 0.56, 95% CI 0.18–1.78; *p* = 0.330; active vs. sham aOR 1.01, 95% CI 0.33–3.07; *p* = 0.983). Similarly, headache did not differ significantly (propofol aOR 0.90, 95% CI 0.42–1.94; *p* = 0.847; active aOR 1.33, 95% CI 0.62–2.86; *p* = 0.560), nor did dizziness (propofol aOR 0.49, 95% CI 0.20–1.22; *p* = 0.125; active aOR 1.83, 95% CI 0.74–4.58; *p* = 0.194). Severity distributions for sore throat and headache were comparable across main effects (both *p* > 0.40, Mann–Whitney), and severe headache (NRS ≥ 7) was rare (Table 4). Intra-operative opioid consumption, converted to morphine milligram equivalents, differed significantly across the four groups (Kruskal–Wallis H = 14.8, *p* = 0.002). Pairwise Mann–Whitney tests showed no difference between active and sham acupuncture within either anesthetic regimen (propofol *p* = 1.000; sevoflurane p = 1.000), whereas propofol-based TIVA required significantly higher MME than sevoflurane across both acupuncture strata (all Bonferroni-adjusted *p* < 0.001) (Table 5).

**Table 4:** Secondary outcomes at 24 h — factorial models and severity distributions.

(A) Any vs. none (factorial logistic models).				
Outcome	Propofol vs. Sevoflurane (aOR, 95% CI)	*p*	Active vs Sham (aOR, 95% CI)	*p*
Sore throat (any vs. none)	0.56 (0.18–1.78)	0.330	1.01 (0.33–3.07)	0.983
Headache (any vs. none)	0.91 (0.42–1.95)	0.799	1.33 (0.62–2.86)	0.469
Dizziness (yes/no)	0.49 (0.20–1.22)	0.125	1.83 (0.73–4.58)	0.194

(B) Severity distributions (Mann–Whitney *p*) and severe headache incidence.			
Outcome/Analysis	Anesthesia *p*	Acupuncture *p*	Severe headache incidence
Sore throat severity distribution	0.509	0.442	—
Headache severity distribution	0.800	0.415	1/65 (1.5%) vs. 1/70 (1.4%); 1/67 (1.5%) vs. 1/68 (1.5%)

Logistic models report adjusted odds ratios (aORs) for main effects from reduced models without the interaction term. Mann–Whitney tests compare ordinal severity distributions between factor levels. Reference levels are sevoflurane and sham acupuncture. For adverse outcomes, aOR < 1 favors propofol and/or active acupuncture. aOR = adjusted odds ratio; CI = confidence interval; NRS = numeric rating scale; IQR = interquartile range.

**Table 5:** Pairwise comparisons of intra-operative opioid consumption (MME, mg).

Comparison	*U*	p_raw	p_Bonferroni
Propofol + Active vs. Propofol + Sham	649.5	0.668	1.000
Propofol + Active vs. Sevoflurane + Active	1044.0	<0.0001	<0.0001
Propofol + Active vs. Sevoflurane + Sham	963.0	<0.0001	<0.0001
Propofol + Sham vs. Sevoflurane + Active	1015.5	<0.0001	<0.0001
Propofol + Sham vs. Sevoflurane + Sham	934.0	<0.0001	<0.0001
Sevoflurane + Active vs. Sevoflurane + Sham	470.5	0.454	1.000

Pairwise group comparisons were performed using the Mann–Whitney *U* test. *P-*values were adjusted for multiple testing using the Bonferroni method.

Omnibus four-arm tests confirmed these patterns. The omnibus χ^2^ test for any PONV was significant (χ^2^(3) = 16.01, *p* = 0.00113); after Holm adjustment, the only significant pairwise contrast was sevoflurane + sham vs. propofol + active (OR 7.50, p_adj = 0.0013). For vomiting ≥1, the omnibus χ^2^ was significant (χ^2^(3) = 12.98, *p* = 0.00469), but no pairwise contrast remained significant after adjustment. Omnibus tests for sore throat, headache, and dizziness were not significant (all *p* ≥ 0.06). Full results are provided in Supplementary Tables S1–S3.

Post-hoc power analyses indicated that, for the primary nausea NRS outcome, power was 68% for the anesthesia main effect, 76% for the acupuncture main effect, and only 5% for the anesthesia × acupuncture interaction (*β* = 0.041, SE = 0.739), showing that the trial was underpowered to detect modest synergy; similarly low interaction power was observed for other outcomes (Supplementary Tables S4). No severe adverse events occurred, and no participant discontinued because of an adverse event.

## 4 Discussion

This randomized controlled trial demonstrated that both propofol-based anesthesia and postoperative acupuncture significantly reduced the incidence of PONV within 24 h after thyroidectomy with intraoperative neuromonitoring. However, the combination of the two interventions did not confer synergistic benefits, indicating additive rather than interactive effects.

The observed incidence of PONV in the sevoflurane groups exceeded that reported in previous thyroidectomy studies (≈44%) (2, 22, 23), reaching up to 60% without acupuncture. This higher rate may reflect the anesthetic adjustments required for IONM, including low-dose cisatracurium and higher opioid administration (24, 25). In this trial, intra-operative opioid consumption, expressed as morphine milligram equivalents, was significantly higher with propofol-based TIVA than with sevoflurane anesthesia, whereas acupuncture did not alter opioid requirements. This suggests that the antiemetic benefit of acupuncture is independent of opioid sparing, and may instead relate to modulation of vagal activity, serotonergic signaling, *β*-endorphin release or central emetic pathways, consistent with mechanisms proposed in previous experimental studies. These multi-target mechanisms help explain why acupuncture remained effective in reducing PONV even under propofol anesthesia, despite higher opioid consumption. Moreover, despite higher opioid use, propofol anesthesia still reduced the incidence of PONV, indicating that its intrinsic antiemetic effect outweighed the potential emetogenic impact of increased opioid exposure.

As a safe non-pharmacological intervention, acupuncture has increasingly been integrated into multimodal therapy (26–28). Previous studies have shown that bilateral Zusanli (ST36) acupuncture combined with ondansetron significantly reduced PONV in patients undergoing laparoscopic surgery (29). Yan et al. also found that combined acupuncture at Neiguan (PC6), Hegu, Zusanli (ST36), and Sanyinjiao was more effective than ondansetron alone in preventing postoperative nausea in high-risk patients (30). Our findings extend this evidence by confirming that acupuncture remains beneficial even alongside propofol-based anesthesia, although without synergy.

Propofol’s antiemetic effects are well recognized and may involve multiple mechanisms, including enhancement of GABA_A receptor activity, suppression of neuronal excitability in the nucleus tractus solitarius and chemoreceptor trigger zone, modulation of 5-HT3 and dopamine D2 signaling, and anti-inflammatory actions (31). Our finding that propofol reduced postoperative dizziness aligns with prior studies (32).

Acupuncture’s anti-PONV effects may involve the modulation of central and peripheral 5-HT levels, enhancement of vagal tone, and promotion of *β*-endorphin release in cerebrospinal fluid. Stimulation at PC6 has been shown to modulate vagal activity and gastric slow waves, regulate serotonergic signaling, and promote β-endorphin release in cerebrospinal fluid, thereby attenuating nausea and improving gastrointestinal motility (15, 17). ST36 complements PC6 by enhancing vagal tone and HRV, while GB20 targets vestibular–occipital circuits implicated in postoperative dizziness and headache. Prior studies combining acupuncture with antiemetics such as ondansetron demonstrated additive efficacy (10, 29). These multi-target mechanisms explain why acupuncture remained effective even in the presence of propofol-based anesthesia.

However, in this factorial RCT, the anesthesia × acupuncture interaction for the PONV endpoints was not significant (e.g., any PONV, Wald *p* = 0.897), indicating additive rather than synergistic effects. This pattern is biologically plausible: propofol-based TIVA already lowers baseline emetogenicity through overlapping antiemetic pathways and by avoiding volatile agents, creating a ceiling once risk is suppressed (7). Acupuncture can reduce PONV and modulate vagal/gastrointestinal signaling (e.g., PC6 stimulation), but the added value on top of effective anesthesia strategies is variable (15, 17). Moreover, propofol may attenuate stimulus-evoked central responses, potentially blunting acupuncture-evoked neural effects (33). Finally, acupuncture is likely dose-dependent (intensity, duration, frequency) (34). Consistent with these mechanisms, post-hoc power for the interaction was low across outcomes (~5–15%; peak 34% for dizziness), implying that only very large interaction effects (interaction OR ≈ 0.07–0.16 or 6–15) would have been detectable; our single, brief perioperative session may have provided insufficient activation. Future trials should test pre-operative initiation, longer or repeated sessions, and standardized stimulation parameters, and be *a priori* powered for an interaction (35).

Early recovery, assessed with a 0–70 short-form score, did not differ between groups (36, 37). The results revealed no significant differences between acupuncture and propofol in improving sleep quality or alleviating sore throat. This could be related to the specific nature of thyroid surgery, including use of large electrophysiological monitoring tubes and required neck hyperextension, which could exacerbate postoperative throat discomfort. In addition, acupuncture was performed only once, and the limited stimulation intensity and frequency may not have been sufficient to yield therapeutic effects within such a short period. The choice of recovery assessment tools may also influence outcomes.

This study has several unique strengths. The active acupuncture procedures were consistently performed by a single experienced acupuncturist, who has extensive clinical experience in managing postoperative gastrointestinal dysfunction (38). Standardized manipulation techniques were applied to ensure consistent stimulation intensity across participants. Additionally, a validated sham acupuncture control was implemented to minimize performance and detection bias.

Several limitations should be acknowledged. First, this was a single-center factorial RCT conducted under uniform IONM workflows with one acupuncturist, which may limit external validity. Routine antiemetic prophylaxis was omitted primarily to isolate the effects of the interventions; local practice constraints, including limited insurance coverage and cost, also contributed. This approach did not fully align with guideline recommendations (7) and represents an important limitation of the trial. Second, the trial was powered for main effects rather than interaction, and the anesthesia × acupuncture interaction estimate was imprecise, so modest synergistic effects cannot be excluded. Third, early recovery was assessed using a 0–70 short form that showed a 24-h ceiling, potentially attenuating differences; future studies could employ more comprehensive tools such as the QoR-40 or Pittsburgh Sleep Quality Index. Fourth, outcomes were assessed to 24 h only, whereas a substantial proportion of PONV occurs within 48 h (39). Fifth, despite rigorous blinding, placebo and expectancy effects cannot be entirely ruled out (15). Finally, the acupuncture protocol involved a single, brief perioperative session; as efficacy is dose- and timing-dependent, preoperative initiation, longer duration, or repeated stimulation may yield stronger effects (34).

In conclusion, this study demonstrated that both acupuncture and propofol anesthesia are effective in reducing PONV. However, their combined application did not demonstrate synergistic advantages, potentially due to differences in intervention mechanisms, limited stimulation frequency, or individual variability. Future research should focus on optimizing acupuncture timing (pre- vs. postoperative), frequency, and intensity, while integrating serological, physiological, and neuroimaging studies to elucidate mechanisms. Additionally, multicenter large-sample randomized trials are needed to confirm the applicability and cost-effectiveness of this approach across different surgeries and populations.

## 5 Conclusion

Acupuncture and propofol-based anesthesia each reduced the incidence of PONV in patients undergoing thyroidectomy with intraoperative neuromonitoring. However, their combination did not demonstrate superiority over either intervention alone.

## Data Availability

The original contributions presented in the study are included in the article/Supplementary material, further inquiries can be directed to the corresponding author/s.
