# Supplementary material for: Clinical trial effects of acupuncture combined with different general anesthesia on postoperative nausea and vomiting and early recovery after thyroidectomy with intra-operative neuromonitoring: a randomized controlled trial
Source: Front Med (Lausanne). 2025 Oct 16;12:1650403. doi: 10.3389/fmed.2025.1650403 (PMC12571839; doi:10.3389/fmed.2025.1650403)
Supplement: Supplementary file 1 [file Data_Sheet_1.docx]

**Supplementary Table S_STRICTA. Completed STRICTA 2010 checklist for the acupuncture intervention**

| **Item** | **Description** |
| --- | --- |
| 1. Rationale | Acupoints PC6, ST36, GB20 selected for mechanisms (antiemetic, gastrointestinal regulation, dizziness relief) and perioperative feasibility. |
| 2. Needling details | Sterile 0.25 mm needles; PC6 10–20 mm vertical; ST36 20–30 mm vertical; GB20 10–15 mm oblique antero-medial (not exceeding 15–20 mm). De-qi elicited. Mild manual stimulation 10–15 s every 10 min. |
| 3. Regimen | Single perioperative session; 25–30 min needle retention spanning emergence; intensity below movement threshold. |
| 4. Other components | Standardized patient instruction scripts; skin antisepsis before needling; prespecified stopping rules. |
| 5. Practitioner background | Credentialed acupuncturists and anesthesiology team, with defined training requirements. |
| 6. Control/comparator | Sham acupuncture procedures (non-penetrating blunt needles) and blinding of patients/outcome assessors. |
| 7. Replicability | A TIDieR checklist accompanies the STRICTA table to facilitate replication. |

Items covered: (1) Rationale (PC6–ST36–GB20, mechanisms and perioperative feasibility); (2) Needling details (sterile 0.25 mm needles; PC6 10–20 mm ⟂; ST36 20–30 mm ⟂; GB20 10–15 mm oblique antero-medial, not exceeding 15–20 mm; de-qi elicited; mild manual stimulation 10–15 s every 10 min); (3) Regimen (single peri-operative session; 25–30 min retention spanning emergence; intensity below movement threshold); (4) Other components (standardized scripts; antisepsis; stopping rules); (5) Practitioner background (credentialed acupuncturists/anaesthesia team with defined training); (6) Control/comparator (sham procedures and blinding). A TIDieR checklist accompanies the STRICTA table to facilitate replication.

**Supplementary Table Sx. 0–70 Short‑Form Early Recovery Score (derived from seven QoR‑15 items)**

| **Domain (EN)** | **Item (EN, paraphrased)** | **Scale / Anchors** | **Scoring** | **QoR‑15 domain mapping / Notes** |
| --- | --- | --- | --- | --- |
| Breathing comfort | Breathing feels comfortable and easy | 0 = very uncomfortable  10 = completely comfortable | 0–10  (higher better) | Physical comfort |
| Appetite | Appetite is adequate | 0 = none  10 = normal appetite | 0–10  (higher better) | Physical comfort / GI symptoms |
| Energy | I feel energetic and able to get on with usual activities | 0 = extremely tired  10 = full of energy | 0–10  (higher better) | Physical independence |
| Sleep quality | Sleep quality since surgery | 0 = very poor  10 = excellent | 0–10  (higher better) | Physical comfort |
| Self‑care / hygiene | Able to wash/groom independently | 0 = unable  10 = completely independent | 0–10  (higher better) | Physical independence |
| Communication | Able to talk normally | 0 = severely limited  10 = normal | 0–10  (higher better) | Physical independence / throat-related comfort |
| Sadness / depression | Feeling sad or depressed | 0 = extremely  10 = not at all | Reverse‑code to 0–10 (higher better) | Emotional state (reverse‑scored) |

Abbreviations: GI = gastrointestinal. Scoring: seven items on 0–10 Likert scales are summed to a total of 0–70; higher scores indicate better recovery. Items are adapted from QoR‑15 domains; this instrument is not the QoR‑15.

**Supplementary Table S1.** Early recovery score (0–70): omnibus ANOVA and pairwise comparisons

| Group | n | Mean ± SD | Median [IQR] |
| --- | --- | --- | --- |
| Sevo + Sham | 32 | 60.41 ± 7.27 | 62 [55–66] |
| Sevo + Active | 33 | 62.42 ± 5.68 | 62 [58–68] |
| Propofol + Sham | 35 | 62.46 ± 5.99 | 64 [60–67] |
| Propofol + Active | 35 | 63.17 ± 6.43 | 64 [62–68] |

Omnibus ANOVA across four arms: F(3,131) = 1.152, p = 0.331.

**Supplementary Table S2.** PONV severity (ordinal 0–3): Kruskal–Wallis and pairwise comparisons

| **Group** | **Severity 0** | **Severity 1** | **Severity 2** | **Severity 3** |
| --- | --- | --- | --- | --- |
| Sevo + Sham | 25.0% | 53.1% | 18.8% | 3.1% |
| Sevo + Active | 60.6% | 33.3% | 6.1% | 0% |
| Propofol + Sham | 54.3% | 31.4% | 14.3% | 0% |
| Propofol + Active | 82.9% | 14.3% | 2.9% | 0% |

Kruskal–Wallis across four arms: H = 23.40, p = 0.00003.

**Supplementary Table S3.** Binary outcomes: pairwise Fisher’s exact tests

| **Outcome** | **Comparison** | **n1** | **n2** | **Cases1** | **Cases2** | **OR** | **95% CI low** | **95% CI high** | **p (Holm)** |
| --- | --- | --- | --- | --- | --- | --- | --- | --- | --- |
| Any PONV | Sevo+Sham vs Sevo+Active | 32 | 33 | 24 | 20 | 1.90 | 0.67 | 5.37 | 0.648 |
| Any PONV | Sevo+Sham vs Propofol+Sham | 32 | 35 | 24 | 16 | 3.41 | 1.23 | 9.44 | 0.097 |

**Supplementary Table S4. Post-hoc power for factorial terms**

| **Outcome** | **β (X1)** | **SE (X1)** | **Power_X1** | **β (X2)** | **SE (X2)** | **Power_X2** | **β (X1×X2)** | **SE (X1×X2)** | **Power_interaction** | **80%-detectable OR** |
| --- | --- | --- | --- | --- | --- | --- | --- | --- | --- | --- |
| Sore throat | 0.539 | 0.473 | 0.207 | 0.568 | 0.469 | 0.227 | -0.608 | 0.662 | 0.151 | 0.16–6.38 |
| Global ordinal score | 0.357 | 0.473 | 0.117 | -0.268 | 0.464 | 0.089 | 0.242 | 0.655 | 0.066 | 0.16–6.25 |
| Nausea NRS | -1.162 | 0.477 | 0.682 | 1.490 | 0.560 | 0.758 | 0.041 | 0.739 | 0.050 | 0.13–7.92 |

Method: Wald noncentral χ² (α=0.05). X1 = propofol vs sevoflurane; X2 = active vs sham acupuncture.
